# Effects of Adding Extracorporeal Shockwave Therapy (ESWT) to Platelet-Rich Plasma (PRP) among Patients with Rotator Cuff Partial Tear: A Prospective Randomized Comparative Study

**DOI:** 10.3390/jpm14010083

**Published:** 2024-01-10

**Authors:** Shu-Jui Kuo, Yu-Hsiang Su, Shih-Chan Hsu, Po-Hua Huang, Chia-Chun Hsia, Chin-Yi Liao, Sung-Hsiung Chen, Re-Wen Wu, Chieh-Cheng Hsu, Yen-Chun Lai, De-Yi Liu, Nien-En Ku, Jui-Feng Chen, Jih-Yang Ko

**Affiliations:** 1 School of Medicine, China Medical University, Taichung 404328, Taiwan; b90401073@gmail.com (S.-J.K.); jamieee0424@gmail.com (S.-C.H.); deyil845@gmail.com (D.-Y.L.); nicheerrr1007@gmail.com (N.-E.K.); dillon0501@gmail.com (J.-F.C.); 2 Department of Orthopedic Surgery, China Medical University Hospital, Taichung 404327, Taiwan; 3 Department of Education, China Medical University Hospital, Taichung 404327, Taiwan; johnny210998@gmail.com (Y.-H.S.); jimmyhsia0422@gmail.com (C.-C.H.); 4 Department of Orthopedic Surgery, College of Medicine, Chang Gung University, Kaohsiung Chang Gung Memorial Hospital, Kaohsiung 833401, Taiwan; 881106@cgmh.org.tw (P.-H.H.); nanaya@cgmh.org.tw (C.-Y.L.); chensh@cgmh.org.tw (S.-H.C.); ray4595@cgmh.org.tw (R.-W.W.); t1234@cgmh.org.tw (C.-C.H.); 5 School of Medicine, National Taiwan University, Taipei 100233, Taiwan; lyc20020130@gmail.com; 6 Center for Shockwave Medicine and Tissue Engineering, College of Medicine, Chang Gung University, Kaohsiung Chang Gung Memorial Hospital, Kaohsiung 833401, Taiwan

**Keywords:** rotator cuff tendinopathy, extracorporeal shock wave therapy (ESWT), platelet-rich plasma (PRP), protein S100A8, protein S100A9

## Abstract

A rotator cuff tear is a prevalent ailment affecting the shoulder joint. The clinical efficacy of combined therapy remains uncertain for partial rotator cuff tears. In this study, we integrated extracorporeal shockwave therapy (ESWT) with platelet-rich plasma (PRP) injection, juxtaposed with PRP in isolation. Both cohorts exhibited significant improvements in visual analogue scale (VAS), Constant–Murley score (CMS), degrees of forward flexion, abduction, internal rotation, and external rotation, and the sum of range of motion (SROM) over the six-month assessment period. The application of ESWT in conjunction with PRP exhibited notable additional enhancements in both forward flexion (*p* = 0.033) and abduction (*p* = 0.015) after one month. Furthermore, a substantial augmentation in the range of shoulder motion (SROM) (*p* < 0.001) was observed after six months. We employed isobaric tag for relative and absolute quantitation (iTRAQ) to analyze the differential plasma protein expression in serum samples procured from the two groups after one month. The concentrations of S100A8 (*p* = 0.042) and S100A9 (*p* = 0.034), known to modulate local inflammation, were both lower in the ESWT + PRP cohort. These findings not only underscore the advantages of combined therapy but also illuminate the associated molecular changes.

## 1. Introduction

Rotator cuff (RC) tendinopathy is a prevailing orthopedic ailment, frequently causing discomfort [1,2,3]. The incidence of RC tendinopathy rises with age, with more than half of the population experiencing a lesion by the time they are 60 years old [4]. Older age, male sex, smoking, diabetes, hypertension, and a higher critical shoulder angle were found to be risk factors for RC tears [5].

In comparison to complete tears, the frequency of partial RC tears is reported to be higher, and the majority of complete tears are attributed to partial tears [6]. The prevalence of partial RC tears is substantial. In the general population, partial RC tear ranges from 15% to 32%, and in the dominant shoulder of professional overhead athletes, it can increase to 40% [7]. Partial RC tears are potentially more painful than full-thickness tears, possibly due to the nonphysiological tension created within the remaining intact RC fibers [8]. Conservative modalities predominate in the realm of RC tendinopathy management, notably in the absence of full tears. Injecting platelet-rich plasma (PRP) and using extracorporeal shockwave therapy (ESWT) have gained increasing popularity as treatment options [7]. ESWT, with its diverse mechanisms, partially unfolds its therapeutic potential through a phenomenon referred to as “regenerative rehabilitation”. This involves physically stimulating damaged tissue, thereby amplifying regenerative processes and augmenting the efficacy of therapeutic procedures [9]. ESWT delivers rapidly rising positive pressure impulses ranging from 5 to 120 MPa in approximately 5 ns, followed by a decrease to negative pressure values of −20 MPa at the treatment site [10]. ESWT can induce hypervascularity in the ischemic rotator cuff tendon and may temporarily increase cell membrane permeability, enhancing the entry of treatment molecules into the cells [11]. These key advantages distinguish ESWT from other potential stimulation methods.

Our prior work revealed that ESWT intervention can yield notable enhancements in visual analogue scale (VAS) assessments, muscle power, Constant–Murley scores (CMS), and range of motion (ROM) within six months post treatment among individuals recovering from RC partial tear [12]. Platelet-rich plasma (PRP) injections exhibited superior early outcomes when juxtaposed with corticosteroid injections in patients presenting with RC partial tear [13].

In the current study, our objectives are focused on delineating the additional therapeutic benefit derived from the combination of ESWT with PRP injection therapy in contrast to the isolated PRP injection therapy. It is our supposition that the synergistic embrace of ESWT alongside injection treatment could potentially yield augmented benefits beyond those engendered by PRP injection therapy.

## 2. Materials and Methods

### 2.1. Recruitment of Participants

This investigation secured the approval of the Institutional Review Board of Chang Gung Medical Foundation (protocol code: 202000068B0, date of approval: 2020/02/13). The eligibility and exclusion criteria for our study are comprehensively summarized in Table 1.

The determination of the minimum requisite sample size for the two groups was executed by using the G*Power 3.1.9.2 software (http://www.gpower.hhu.de/en.html (accessed on 1 January 2016)) before the recruitment of participants [14]. The a priori power analysis employed a two-tailed Wilcoxon signed-rank test and Mann–Whitney U test (two groups) to deduce a sample size of no less than 27 for each group, based on a calculated effect size of 0.8, an α level of 0.05, a power of 80%, and an allocation ratio of 1.

The participants were randomized to receive either PRP injection combined with ESWT or PRP injection along with sham ESWT, involving simulated energy transmission, immediately prior to the intervention. The randomization was conducted using a computer-generated list, and the allocation was concealed within a series of numbered envelopes. The participants and the follow-up examiners were all kept unaware of the treatment assignment.

### 2.2. Imaging Diagnosis of Rotator Cuff (RC) Lesions

The diagnostic assessment of RC partial tear involved the agreement of both a musculoskeletal radiologist proficient in the interpretation of MRI for the shoulder joint and the corresponding author. Regarding the MRI, tendinosis was revealed by augmented intratendinous signal intensity on T2-weighted images without complete disruption. Partial-thickness tearing manifested as a hyperintense fluid or fluid-like signal intensity extending into the tendon on T2-weighted images. Full-thickness tears were diagnosed if hyperintense fluid or fluid-like signal intensity permeated the complete thickness of the disrupted RC tendon on T2-weighted images.

### 2.3. Platelet-Rich Plasma (PRP) Injection

Details of the PRP injection therapy are provided herein. Ten milliliters of venous blood was extracted from each participant, and the extracted blood underwent processing using the RegenKit THT system (RegenLab SA, Le Mont-sur-Lausanne, Switzerland) [15]. The subacromial injections were administered by the corresponding author, a specialist with over two decades of experience in shoulder surgery. The injection procedure was effectuated by adopting a posterolateral approach, positioned approximately 1.5 finger’s breadths below the posterolateral corner of the acromion, without the utilization of local anesthesia. The needle was adeptly maneuvered along the superior border of the rotator cuff into the subacromial space. If the needle tip came into contact with the undersurface of the acromion, slight withdrawal of the needle facilitated the smooth administration of the content.

### 2.4. Extracorporeal Shockwave (ESWT) Therapy

Subjects receiving genuine ESWT were exposed to the delivery of 3000 shockwave impulses calibrated at 24 kV (energy flux density = 0.32 mJ/mm^2^), during a single hour-long session one week after PRP injection [12]. These therapeutic shockwaves emanated from the Orthospec™ Extracorporeal Shockwave device (Orthospec™, Medispec, Yehud, Israel). This therapeutic protocol was expertly overseen by a certified specialist in an outpatient setting. The shockwaves were carefully directed, with precise clinical focus on the rotator interval (one finger’s breadth laterally and superiorly to the coracoid process) and the rotator cable (thick fibrous bundle transmitting applied forces to RC). The juxtaposition of the shockwave tube and the skin was facilitated by the application of surgical lubricant, while the usage of local anesthetics was decidedly eschewed. In contrast, subjects enrolled within the sham ESWT cohort underwent a simulated intervention. In the simulated endeavor, the device was employed without the silicone pad on the stand-off device. Although the audible manifestations of the shockwaves and a tactile tingling sensation were experienced by the participants, the actual energy transmission was altogether withheld. The entire therapeutic procedure was conducted under the vigilant monitoring of vital signs, and any potential sensations of discomfort were dutifully observed and documented. Post intervention, the treated regions underwent meticulous inspection to ascertain the presence of any local manifestations such as swelling, ecchymosis, or hematoma. Following the completion of the intervention, the baseline regimen encompassing activity modification and/or physiotherapy was judiciously sustained. As the aftereffects of the intervention unfolded, the participants were duly initiated into a regimen of gentle pendulum exercises and meticulously guided assisted shoulder movements, involving elevation, external rotation, and internal rotation.

Patients were advised to limit their analgesic intake to a daily dosage of 1000 mg acetaminophen and abstain from any anti-inflammatory agents after the intervention. In anticipation of each assessment, patients dutifully abstained from the use of pain medication for three days. In cases where enduring and severe shoulder discomfort or loss of function persisted, surgical intervention was recommended.

### 2.5. Clinical Assessments

The clinical parameters, which included the visual analogue scale (VAS), muscle power for shoulder abduction, Constant–Murley score (CMS), and range of motion (ROM) of the shoulder, were evaluated at 1 week (1 W), 1 month (1 M), 3 months (3 M), and 6 months (6 M) following ESWT or sham ESWT. The VAS serves as a pain measurement scale, where 0 indicates absence of pain and 10 signifies unbearable pain. Muscle power for shoulder abduction was evaluated by measuring the maximal isometric contraction of the abductor muscles. This assessment utilized a handheld Baseline 250 hydraulic push–pull dynamometer (Baseline Corporation, Irvington, NY, USA) with the shoulder positioned at 45° abduction, the elbow at 90° flexion, and the arm internally rotated without torso stabilization. The Constant–Murley score (CMS) is a standardized scale for evaluating shoulder function, with a maximum score of 100 denoting optimal shoulder function. This scoring system has been employed in numerous studies for evaluating shoulder-related outcomes [4,16,17,18]. Shoulder ROM was evaluated with the patient seated. A goniometer was utilized to measure the extent to which the patient could passively forward flex or abduct the shoulder. External rotation and internal rotation of the shoulders were assessed with the patient’s arm in a resting position and at a 45° flexion position, respectively. The sum of range of motion (SROM) was calculated by summing the measured ROM values. The minimal clinically important difference (MCID) for VAS and CMS was set at 2 and 10, respectively [19]. The patient acceptable symptom state (PASS) for VAS and CMS was determined as 0.9 and 80, respectively [19].

### 2.6. Assessments of Isobaric Tag for Relative and Absolute Quantitation (iTRAQ)

Isobaric tag for relative and absolute quantitation (iTRAQ) constitutes a mass spectrometry-based proteomic quantification technique that employs the derivatization of primary amino groups within intact proteins, along with isobaric tags assigned to distinct peptide fragments [20,21,22,23,24,25]. The iTRAQ methodology affords the opportunity to simultaneously screen up to eight samples for differentially expressed proteins with exceptional sensitivity and specificity [26]. In this study, we harnessed the potency of iTRAQ to comprehensively scrutinize serum samples exhibiting disparate levels consequent to the assigned interventions. Employing the iTRAQ gel-free proteomics technology, we elucidated the intricate protein profiles inherent to the serum samples [27]. To abrogate any confounding signals originating from background proteins, we employed the Pierce Top 12 Abundant Protein Depletion Spin Columns (85165, Thermo) for the purpose of extracting high-abundance proteins from the serum samples. Subsequently, we prepared the serum samples by utilizing the iTRAQ Reagents Multiplex Kit (4352135, SCIEX), thereby ensuring that the peptides therein were appropriately labelled with tags of diverse molecular weights. These adorned samples were then analyzed by the LC/Q-Exactive Orbitrap MS (Thermo) for a duration of 24 h. The resultant raw data underwent further meticulous analysis by the Proteome Discoverer v2.4 (Thermo), with reference to the MASCOT 2.5 database (Matrix Science).

### 2.7. Statistical Analysis

For non-parametric within-group comparisons, the Friedman test was utilized for assessing repeated measurements of continuous variables, and the Wilcoxon signed-rank test was applied for post hoc analysis [14,28]. For parametric within-group comparisons, the repeated measures ANOVA test was employed for the assessment of repeated within-group measurements for continuous variables, and the Fisher’s least significant difference (LSD) test was applied for post hoc analysis. To facilitate a nuanced inter-group comparison, the chi-square and Mann–Whitney U (non-parametric)/Student’s t (parametric) tests were appropriately employed for categorical and continuous variables, respectively.

## 3. Results

**Figure 1:**
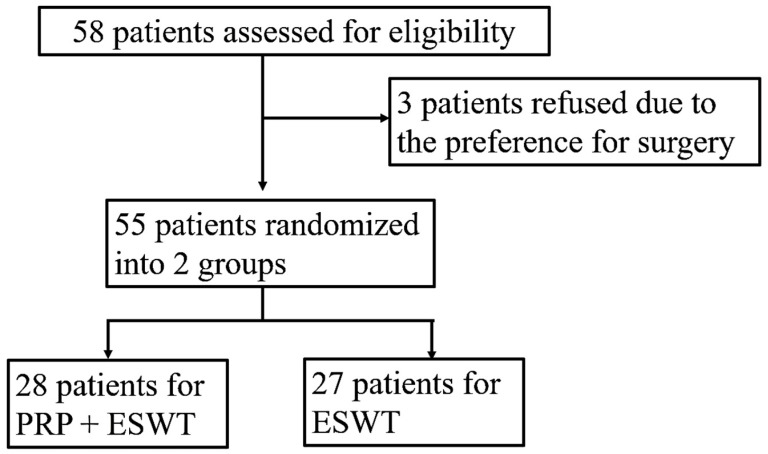
The flow chart for the randomization process.

There were 28 and 27 patients in the PRP + ESWT and PRP groups, respectively. The randomization process is illustrated in Figure 1. The demographic profiles are shown in Table 2.

**Figure 2:**
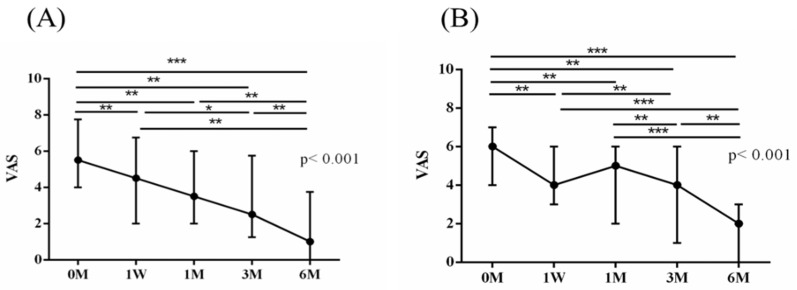
The alterations in visual analogue scale (VAS) over the course of this study. (**A**) PRP + ESWT group; (**B**) PRP group. The error bars in the figure indicate the median and the 25th and 75th percentiles of the data (* *p* < 0.05, ** *p* < 0.01, and *** *p* < 0.001).

**Figure 3:**
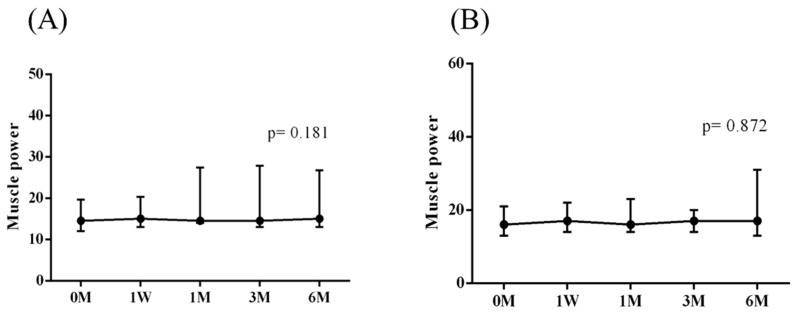
The alterations in muscle power of abduction over the course of this study. (**A**) PRP + ESWT group; (**B**) PRP group. The error bars in the figure indicate the median and the 25th and 75th percentiles of the data.

**Figure 4:**
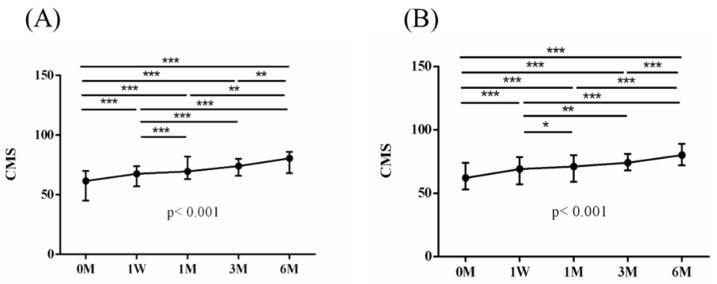
The alterations in Constant–Murley score (CMS) over the course of this study. (**A**) PRP + ESWT group; (**B**) PRP group. The error bars in the figure indicate the median and the 25th and 75th percentiles of the data (* *p* < 0.05, ** *p* < 0.01, and *** *p* < 0.001).

**Figure 5:**
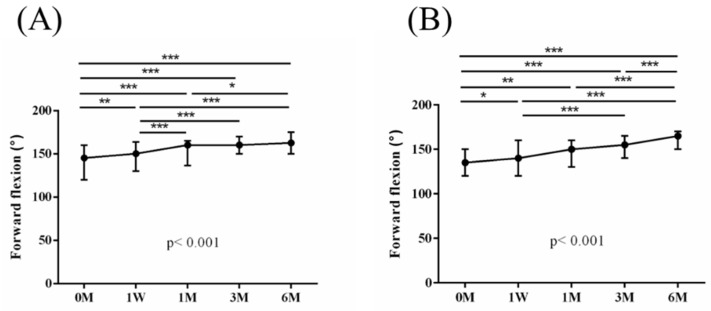
The alterations in the degrees of forward flexion over the course of this study. (**A**) PRP + ESWT group; (**B**) PRP group. The error bars in the figure indicate the median and the 25th and 75th percentiles of the data (* *p* < 0.05, ** *p* < 0.01, and *** *p* < 0.001).

**Figure 6:**
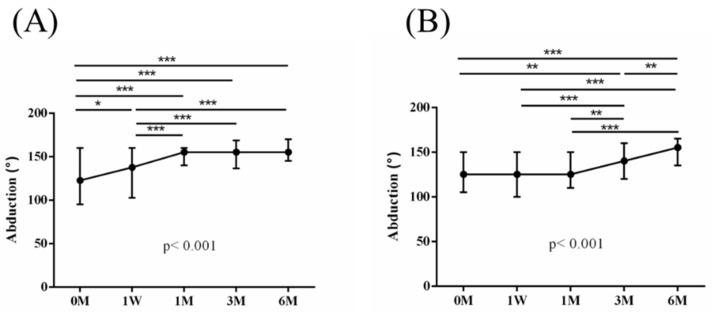
The alterations in the degrees of abduction over the course of this study. (**A**) PRP + ESWT group; (**B**) PRP group. The error bars in the figure indicate the median and the 25th and 75th percentiles of the data (* *p* < 0.05, ** *p* < 0.01, and *** *p* < 0.001).

**Figure 7:**
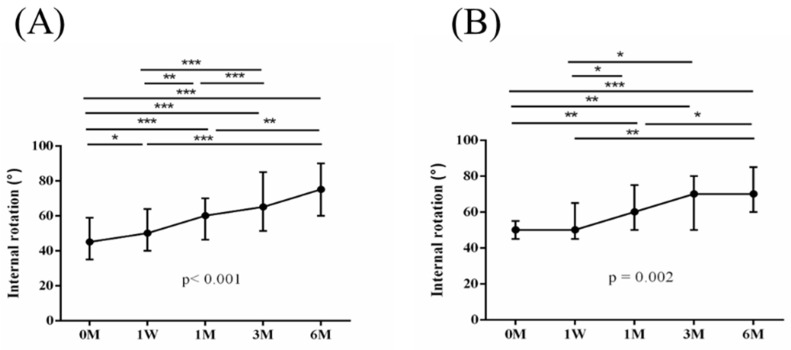
The alterations in the degrees of internal rotation over the course of this study. (**A**) PRP + ESWT group; (**B**) PRP group. The error bars in the figure indicate the median and the 25th and 75th percentiles of the data (* *p* < 0.05, ** *p* < 0.01, and *** *p* < 0.001).

**Figure 8:**
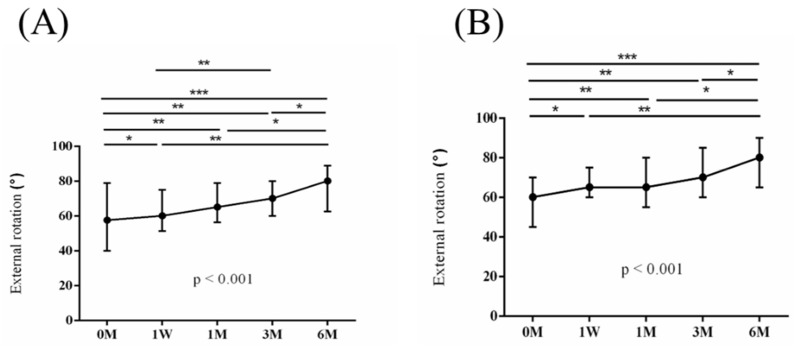
The alterations in the degrees of external rotation over the course of this study. (**A**) PRP + ESWT group; (**B**) PRP group. The error bars in the figure indicate the median and the 25th and 75th percentiles of the data (* *p* < 0.05, ** *p* < 0.01, and *** *p* < 0.001).

**Figure 9:**
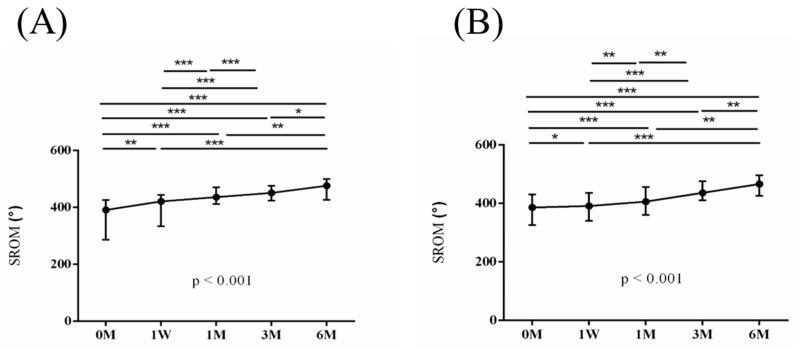
The alterations in the degrees of the sum of range of motion (SROM) over the course of this study. (**A**) PRP + ESWT group; (**B**) PRP group. The error bars in the figure indicate the median and the 25th and 75th percentiles of the data (* *p* < 0.05, ** *p* < 0.01, and *** *p* < 0.001).

The intra-group alterations in visual analogue scale (VAS), muscle power of abduction, and the Constant–Murley score (CMS) over the course of this study are depicted in Figure 2, Figure 3 and Figure 4. Similarly, the intra-group variations in the degrees (°) of forward flexion, abduction, internal rotation, external rotation, and the sum of range of motion (SROM) throughout the study duration are illustrated in Figure 5, Figure 6, Figure 7, Figure 8 and Figure 9.

The baseline measurements of VAS, muscle power of abduction, CMS, and the degrees of forward flexion, abduction, internal rotation, external rotation, and the SROM were similar among the two groups. No additional benefits of ESWT were observed in terms of visual analogue scale (VAS), muscle power of abduction, and Constant–Murley score (CMS) throughout the study period from baseline to six months after intervention (Table 3 and Table 4). The supplementary advantages of ESWT in conjunction with PRP injection became evident in forward flexion (*p* = 0.033) and abduction (*p* = 0.015) after one month of intervention, and in SROM (*p* < 0.001) after six months of intervention (Table 5). The outcomes of inter-group comparisons are outlined in Table 3, Table 4 and Table 5.

The iTRAQ gel-free proteomic technology was employed to detect differential plasma protein levels across distinct serum samples. Samples were collected, including one from PRP and another from PRP + ESWT, 1 month after intervention. By applying identification parameters of a false determinate rate <0.01 for protein and peptide identification and requiring protein identification with at least one unique peptide, 688 proteins were assessed and quantified. Abundance quantification of these proteins was further analyzed by using Partek [25]. With parameters set at *p*-value < 0.05 and variation >1.25, 15 proteins exhibited differential abundances between the two groups. Notably, several of these proteins were linked to inflammation. These findings suggest that PRP + ESWT may yield superior treatment efficacy compared to PRP alone, possibly by counteracting the inflammation-promoting effect of standalone PRP treatment.

Among the proteins displayed in Table 6, we are especially interested in S100A9. S100A8 and S100A9 are well-known inflammatory plasma proteins involved in the inflammatory disorders, including osteoarthritis [29,30]. The serum samples were procured from the participants within the two cohorts one month post intervention. Notably, the serum concentrations of S100A8 and S100A9 were markedly diminished among the patients in the PRP+ ESWT group (Table 7).

## 4. Discussion

The potential therapeutic benefits of ESWT for patients with incomplete RC tears have been previously investigated. In our prior prospective research, we observed that ESWT, when compared to a sham treatment, exhibited significant improvements in VAS, muscle power, CMS, and ROM in both the 6-month and 12-month post-intervention assessments. This underscores the therapeutic efficacy of ESWT in addressing incomplete RC tears associated with shoulder stiffness [12]. In Chou et al.’s retrospective study, ESWT yielded a satisfaction rate surpassing 50% among patients with incomplete RC tears. Additionally, 53.8% of athletes returned to their prior activity levels, a result comparable to surgery [31].

PRP is a concentrated solution of platelets that is rich in growth factors, showing the ability to facilitate angiogenesis, neuroprotection, neural regeneration, regulation of inflammation with broad therapeutic applications [17,32,33,34,35,36,37,38,39,40,41,42]. Comparative studies have shown the clinical advantages of PRP injection for patients with incomplete RC tears. Ahmed Shams et al. conducted a study comparing PRP and corticosteroid injections in 40 patients. After 12 weeks, they found a statistically significant advantage in the PRP group over the corticosteroid group in terms of VAS, American Shoulder and Elbow Surgeons Standardized Shoulder Assessment Form (ASES), CMS, and Simple Shoulder Test (SST) [13]. Thanathep Tanpowpong et al. showed that PRP effectively reduces the size of supraspinatus tendon tears, surpassing corticosteroid [43]. Lutz von Wehren observed significant improvements in shoulder scores for both PRP and cortisone groups. Assessments at 12 weeks, including VAS, ASES, SST, and CMS, favored the PRP group [44]. Damjanov et al. showed that PRP significantly improved shoulder pain, surpassing glucocorticoid effects, with sustained benefits at 4 and 24 weeks. PRP patients also exhibited significantly greater CMS improvements at 24 weeks, and none reported adverse events, unlike the betamethasone group (*n* = 8) [45]. Aylin Sari et al. divided 129 patients into PRP, corticosteroid, prolotherapy, and lidocaine groups. In the PRP group at week 24, VAS and western Ontario rotator cuff (WORC) scores were notably lower than the corticosteroid group [46]. Cai et al. found significant differences in CMS, VAS, and ASES scores at 12 months in the SH (sodium hyaluronate) + PRP group. MRI results indicated a notable reduction in tear size, especially in the SH + PRP group. The study concludes that PRP injection effectively heals partial RC tears, with SH + PRP providing superior clinical outcomes compared to SH or PRP alone [47]. Collectively, these studies provide evidence that PRP surpasses other injection therapies as the preferred modality for treating incomplete RC tears.

The preceding studies underscore the therapeutic merits of ESWT and PRP as standalone treatments for incomplete RC tears. Our research extends this literature by revealing the added benefits of combining ESWT with PRP for patients with incomplete RC tears. Notably, 1 month post intervention, there were significant improvements in forward flexion (*p* = 0.033) and abduction (*p* = 0.015). Additionally, sustained enhancement in SROM (*p* < 0.001) was observed after 6 months of intervention, emphasizing the synergistic advantages of ESWT with PRP for shoulder ROM.

Besides improved shoulder ROM, we aim to elucidate the mechanistic underpinnings of this additional benefit. In our study, the distinct serum protein expression pattern among patients undergoing ESWT + PRP or PRP alone was detected through the iTRAQ assay one month post intervention. Reduced serum levels of S100A8 and S100A9 were confirmed through ELISA in patients undergoing ESWT + PRP. S100A8 and S100A9 are noteworthy alarmins—endogenous immune-activating proteins released into the extracellular milieu following tissue damage to initiate and augment inflammatory responses [48,49,50,51].

Both S100A8 and S100A9 exhibit chemotactic properties towards monocytes and are linked to myeloid cell maturation. They have the potential to induce both pro- and anti-inflammatory effects by modulating the cytokine profile through interaction with pattern recognition receptors (PRRs). This chemotaxis and cytokine modulation are particularly prominent in the early stages of tendinopathy, establishing a mechanistic link between S100A8 and S100A9 proteins and RC tendinopathy development. Exposing primary human tenocytes to exogenous S100A8/A9 resulted in a significant increase in the release of IL-6, IL-8, CCL2, CCL20, and CXCL10 proteins. This implies that S100A8/S100A9 modulates the inflammatory profile through a positive feedback mechanism, involving increased recruitment of leukocytes and the release of pro-inflammatory cytokines from tenocytes, sustaining the inflammatory response in the early stages of tendinopathy [52]. Our research provides novel evidence indicating an association between supplementary ESWT and an anti-inflammatory effect, as evidenced by reduced systemic levels of S100A8/A9.

Our study has certain limitations. Firstly, not all of the markers of differential expression as determined by iTRAQ were quantified by ELISA. The decision to focus our investigation on proteins S100A8 and S100A9 was rooted in our review of the existing literature. Secondly, the present study did not establish a direct correlation between the serum levels of S100A8/S100A9 and the functional assessments. This is an avenue that warrants exploration in subsequent research. Thirdly, our study did not provide conclusive evidence of differential image improvement, which highlights the need for further inquiry in this domain. Fourthly, ultrasonic guidance was not utilized during the PRP injection, as unguided injections into the subacromial bursa are considered less optimal compared to ultrasound-guided procedures [53]. Fifthly, the specific mode of ESWT application was referenced in our previous publication, and whether it is possible to use stimuli of lower or higher intensity warrants further investigation. Finally, shoulder abduction and forward flexion may occur due to deltoid contraction. We cannot rule out the possibility that the noted enhancements in shoulder abduction and forward flexion might be attributed to the application of ESWT on the potential deltoid contracture site rather than the site of the RC partial tear. Despite these limitations, our study represents a pioneering effort in demonstrating the advantages of combining ESWT and PRP treatments over PRP alone. We have shed light on the distinct serum levels of protein S100A8 and protein S100A9 between the two groups. These findings offer clinicians valuable insight into the potential benefits of employing a combined approach of ESWT and PRP injection for the treatment of partial RC tears.

## 5. Conclusions

The combination of ESWT with PRP injection potentially transcends the advantages afforded by PRP injection monotherapy in the context of RC partial tear, as evidenced by the amelioration of clinical assessment parameters and the modulation of serum inflammatory markers. We hope that these data can prompt clinicians to consider the therapeutic effects of combining ESWT with PRP injection therapy for RC partial tears.

## Figures and Tables

**Table 1:** The inclusion and exclusion criteria for this study.

Inclusion criteria	Exclusion criteria
VAS score surpassing 3	Rheumatic diseases
Positive impingement sign	Glenohumeral osteoarthritis
Pain during Hawkins’ test or empty can test	Full-thickness RC tear
MRI evidence of a supraspinatus lesion without a complete tear	Fractures
Pain and/or stiffness resistance to modifications in physical activity and/or therapeutic interventions under professional therapists for at least 3 months	Infections
Aged between 35 and 80 years	Neoplasms
	Pregnancy
	Subacromial injections within the preceding 3 weeks
	Not submitting valid written informed consent

**Table 2:** Baseline characteristics for the two groups.

	PRP + ESWT	PRP	*p*-Value
Male/female	8/20	13/14	0.135
Age	57.5 (53.0, 66.5)	57.0 (51.5, 64.5)	0.826
Left/right	11/17	11/16	0.912
Body mass index	24.2 (23.0, 26.8)	24.4 (22.7, 28.4)	0.976
Medical comorbidities ^a^	10	14	0.228
VAS	5.5 (4.5,7.5)	6.0 (4.0, 7.0)	0.936
	5.46 ± 2.17	5.37 ± 2.34	
Muscle power (lb)	14.5 (12.0, 20.0)	16.0 (13.5, 20.5)	0.093
	18.20 ± 9.45	21.10 ± 11.78	
CMS	61.5 (45.0, 69.5)	62.0 (53.0, 73.5)	0.412
	59.30 ± 14.20	62.69 ± 12.65	
Forward flexion (°)	145.0 (120.0, 160.0)	135.0 (120.0, 150.0)	0.435
	136.11 ± 26.94	133.70 ± 21.64	
Abduction (°)	122.5 (95.0, 160.0)	125.0 (107.5, 150.0)	0.968
	123.39 ± 32.35	124.63 ± 27.70	
IR (°)	45.0 (35.0, 56.3)	50.0 (45.0, 55.0)	0.162
	46.07 ± 19.64	52.41 ± 17.83	
ER (°)	57.5 (40.0, 76.3)	60.0 (47.5, 70.0)	0.711
	58.93 ± 21.32	61.11 ± 18.15	
SROM (°)	390.0 (288.8, 425.0)	385.0 (327.5, 425.0)	0.992
	365.36 ± 84.11	371.85 ± 67.30	

^a^ Diabetes, coronary artery disease, chronic kidney disease.

**Table 3:** The visual analogue scale (VAS), muscle power of abduction, and Constant–Murley score (CMS) for subjects receiving the assigned interventions.

	PRP + ESWT	PRP	*p*-Value
**Baseline profiles**			
VAS	5.5 (4.5,7.5)	6.0 (4.0, 7.0)	0.936
	5.46 ± 2.17	5.37 ± 2.34	
Muscle power (lb)	14.5 (12.0, 20.0)	16.0 (13.5, 20.5)	0.093
	18.20 ± 9.45	21.10 ± 11.78	
CMS	61.5 (45.0, 69.5)	62.0 (53.0, 73.5)	0.412
	59.30 ± 14.20	62.69 ± 12.65	
**One week**			
VAS	5.5 (5.0, 10.0)	5.0 (5.0, 10.0)	0.976
	4.32 ± 2.44	4.26 ± 2.33	
Muscle power (lb)	15.0 (13.0, 20.4)	17.0 (14.0, 22.0)	0.234
	18.88 ± 9.17	21.64 ± 11.96	
CMS	67.5 (57.0, 73.5)	69.0 (57.0, 78.5)	0.529
	65.77 ± 12.06	67.22 ± 12.61	
**One month**			
VAS	3.5 (2.0, 6.0)	5.0 (2.0, 6.0)	0.787
	3.89 ± 2.40	4.11 ± 2.52	
Muscle power (lb)	14.5 (14.0, 27.0)	16.0 (14.0, 23.0)	0.322
	19.67 ± 9.42	21.55 ± 11.01	
CMS	69.5 (63.0, 81.5)	71.0 (59.0, 80.0)	0.697
	71.29 ± 10.49	69.69 ± 11.37	
**Three months**			
VAS	2.5 (2.0, 5.0)	4.0 (1.0, 6.0)	0.873
	3.57 ± 2.53	3.48 ± 2.59	
Muscle power (lb)	14.5 (13.0, 26.0)	17.0 (14.0, 20.0)	0.313
	19.26 ± 8.89	21.09 ± 11.33	
CMS	74.0 (67.3. 79.4)	74.0 (68.5, 80.5)	0.873
	72.32 ± 12.29	72.04 ± 13.70	
**Six months**			
VAS	1.0 (0.5, 3.5)	2.0 (0.5, 3.0)	0.912
	2.25 ± 2.53	2.04 ± 1.91	
Muscle power (lb)	15.0 (13.0, 26.7)	17.0 (13.5, 28/0)	0.352
	19.68 ± 9.41	22.59 ± 12.65	
CMS	81.0 (71.0, 86.0)	80.0 (72.0, 87.3)	0.719
	76.75 ± 14.27	79.33 ± 11.82	

VAS: visual analogue scale; CMS: Constant–Murley score.

**Table 4:** The number of participants achieving the minimal clinically important difference (MCID) (≥2 for VAS and ≥10 for CMS) and patient acceptable symptom state (PASS) (≥0.9 for VAS and ≥80 for CMS).

	MCID		PASS	
	PRP + ESWT	PRP	PRP + ESWT	PRP
	N = 28	N = 27	N = 28	N = 27
1 week				
VAS	9	10	0	3
CMS	8	4	3	4
1 month				
VAS	14	9	0	4
CMS	17	8	8	7
3 months				
VAS	16	17	1	4
CMS	13	15	7	9
6 months				
VAS	19	21	8	7
CMS	20	22	15	15

**Table 5:** The range of motion (ROM) and the sum of range of motion (SROM) for the subjects receiving the assigned interventions.

	PRP + ESWT	PRP	*p*-Value
**Baseline profiles**			
Forward flexion (°)	145.0 (120.0, 160.0)	135.0 (120.0, 150.0)	0.435
	136.11 ± 26.94	133.70 ± 21.64	
Abduction (°)	122.5 (95.0, 160.0)	125.0 (107.5, 150.0)	0.968
	123.39 ± 32.35	124.63 ± 27.70	
IR (°)	45.0 (35.0, 56.3)	50.0 (45.0, 55.0)	0.162
	46.07 ± 19.64	52.41 ± 17.83	
ER (°)	57.5 (40.0, 76.3)	60.0 (47.5, 70.0)	0.711
	58.93 ± 21.32	61.11 ± 18.15	
SROM (°)	390.0 (288.8, 425.0)	385.0 (327.5, 425.0)	0.992
	365.36 ± 84.11	371.85 ± 67.30	
**One week**			
Forward flexion (°)	150.0 (122.5, 160.0)	140.0 (125.0, 150.0)	0.401
	142.41 ± 23.18	138.70 ± 21.82	
Abduction (°)	135.0 (96.3, 158.8)	125.0 (100.0, 150.0)	0.497
	130.89 ± 29.91	125.74 ± 26.70	
IR (°)	50.0 (35.0, 60.0)	50.0 (45.0, 62.5)	0.478
	52.68 ± 18.83	55.19 ± 14.84	
ER (°)	60.0 (45.0, 75.0)	65.0 (51.5, 75.0)	0.509
	63.57 ± 18.05	66.11 ± 15.46	
SROM (°)	405.0 (330.0, 430.0)	385.0 (337.5, 427.5)	0.728
	390.00 ± 69.34	385.74 ± 59.53	
**One month**			
Forward flexion (°)	160.0 (136.3, 165.0)	150.0 (130.0, 160.0)	0.033
	153.70 ± 15.79	142.22 ± 21.05	
Abduction (°)	155.0 (140.0, 160.0)	125.0 (110.0, 150.0)	0.015
	144.82 ± 24.40	129.44 ± 22.80	
IR (°)	60.0 (46.3, 70.0)	60.0 (50.0, 75.0)	0.787
	60.36 ± 15.15	61.48 ± 18.12	
ER (°)	65.0 (56.3, 78.8)	65.0 (57.5, 80.0)	0.928
	66.79 ± 14.42	67.59 ± 15.95	
SROM (°)	435.0 (411.3, 470.0)	405.0 (362.5, 452.5)	0.089
	425.89 ± 52.49	400.74 ± 60.30	
**Three months**			
Forward flexion (°)	160.0 (150.0, 170.0)	155.0 (142.5, 165.0)	0.267
	156.67 ± 15.32	150.74 ± 20.37	
Abduction (°)	155.0 (138.8, 166.3)	140.0 (120.0, 160.0)	0.091
	149.46 ± 20.74	139.26 ± 22.86	
IR (°)	65.0 (53.8, 85.0)	70.0 (52.5, 80.0)	0.711
	67.50 ± 16.36	65.00 ± 19.46	
ER (°)	70.0 (60.0, 80.0)	70.0 (62.5, 85.0)	0.719
	69.29 ± 15.68	69.63 ± 18.96	
SROM (°)	450.0 (427.5, 475.0)	435.0 (412.5, 472.5)	0.610
	443.39 ± 47.86	424.63 ± 68.65	
**Six months**			
Forward flexion (°)	165.0 (150.0, 175.0)	165.0 (152.5, 170.0)	0.728
	158.89 ± 19.28	159.63 ± 14.87	
Abduction (°)	155.0 (145.0, 170.0)	155.0 (137.5, 165.0)	0.596
	152.14 ± 22.09	148.89 ± 21.94	
IR (°)	75.0 (62.5, 90.0)	70.0 (60.0, 85.0)	0.610
	71.43 ± 19.19	69.63 ± 17.92	
ER (°)	80.0 (70.0, 87.5)	80.0 (67.5, 90.0)	0.897
	73.21 ± 19.40	74.81 ± 16.61	
SROM (°)	475.0 (450.0, 497.5)	465.0 (412.5, 495.0)	< 0.001
	455.36 ± 68.66	452.96 ± 54.07	

IR: internal rotation; ER: external rotation; SROM: sum of range of motion.

**Table 6:** The proteins with differential abundances between the PRP + ESWT and PRP groups.

Protein	PRP + ESWT/PRP Ratio	*p*-Value
Apolipoprotein C4	0.866	<0.001
Adhesion G protein-coupled receptor G6	0.741	0.013
YWHAE	0.842	0.013
Phosphatidylethanolamine-binding protein 4	0.786	0.014
Lactoferrin	0.767	0.021
CD5-like molecule	0.893	0.023
Fermitin family homolog 3	0.914	0.023
Mannan-binding lectin serine protease 1	0.948	0.024
H4 clustered histone 1	1.305	0.024
Paraoxonase 3	1.046	0.027
Apolipoprotein A2	1.047	0.028
Keratin 9	0.443	0.035
S100 calcium-binding protein A9	0.573	0.042
Intercellular adhesion molecule 2	1.110	0.043
Apolipoprotein H	0.972	0.045

YWHAE: tyrosine 3-monooxygenase/tryptophan 5-monooxygenase activation protein epsilon.

**Table 7:** The serum levels of S100A8 and S100A9 1 month after intervention among the two groups.

	PRP + ESWT	PRP	*p*-Value
S100A8	12.53 (3.21, 15.77)	22.45 (10.33, 35.11)	0.042
S100A9	34.32 (20.71. 47.73)	57.67 (51.06, 119.70)	0.034

## Data Availability

The data presented in this study are available on request from the corresponding author.
